# How should we manage patients with interferon-gamma release assay-positive results in rheumatology: assessing the risk and long-term prognosis of tuberculosis

**DOI:** 10.1016/j.ero.2025.10.003

**Published:** 2025-11-05

**Authors:** Shinichi Nogi, Hiroshi Furukawa, Yusuke Yano, Shigeto Tohma, Atsushi Hashimoto, Toshihiro Matsui

**Affiliations:** 1 Department of Rheumatology, National Hospital Organization Sagamihara National Hospital, Sagamihara, Japan; 2 Department of Rheumatology, National Hospital Organization Tokyo National Hospital, Kiyose, Japan; 3 Department of Internal Medicine, Sagami Seikyo Clinic, Sagamihara, Japan

## Abstract

**Objectives:**

Tuberculosis (TB) is a global health problem. Screening for latent TB infection (LTBI) and the administration of preventive treatment in populations at high risk of developing TB are considered crucial. Although patients with rheumatic diseases are at high risk, there are few reports on the screening, prevalence, treatment, and prognosis of LTBI in this setting. In this study, we investigated the characteristics of patients with LTBI in a rheumatology department in Japan.

**Methods:**

We collected interferon-gamma release assay (IGRA) results and patient information from the medical records of patients who attended the rheumatology department at National Hospital Organization Sagamihara National Hospital (Sagamihara, Japan) from 2013 to 2022.

**Results:**

Of 4072 patients tested with IGRA, 157 were positive (3.9%). The rates of IGRA positivity decreased with time (*P* = .00047), whereas the ages of patients with IGRA-positive results increased (*P* = .048). The rates of IGRA positivity were higher in patients born before 1960 than those born thereafter (*P* < .0001). Mycobacterial or imaging tests were performed in almost all IGRA-positive patients (*n* = 154, 98.1%). Although less than one-third of the patients with IGRA-positive results were treated with isoniazid (*n* = 50, 31.8%), there was no development of active TB during the average follow-up period of 4.9 years.

**Conclusions:**

In IGRA-positive patients, it is important to perform mycobacterial and imaging tests, as well as long-term follow-up.

## INTRODUCTION

Tuberculosis (TB) is a major health problem and one of the 3 most prevalent infectious diseases worldwide. According to the World Health Organization, approximately 7.5 million individuals were newly diagnosed with TB in 2022. This is the highest number reported since the World Health Organization began global TB monitoring in 1995. Furthermore, 1.3 million TB-related deaths occurred in 2022 [1]. Additionally, it is estimated that approximately a quarter of the global population has been infected with TB [1]. Screening for latent TB infection (LTBI) and the administration of preventive treatment in populations at high risk of developing TB are considered crucial to prevent progression from LTBI to active TB.

For many years, Japan experienced a high incidence rate of TB. However, in 2021, it was finally designated as a country with a low TB burden. By 2023, the incidence rate of TB in Japan had decreased to 8.1 cases per 100,000 individuals [2]. Nevertheless, this figure remains higher than that observed in other developed countries. Notably, the incidence rate of TB among elderly individuals in Japan is high. In 2023, 10,096 new patients with TB were registered; of those, 70.6% were aged >60 years [2]. In addition to TB, the number of elderly patients with rheumatoid arthritis (RA) (representing inflammatory rheumatic diseases) and the age at onset of RA have been increasing [3]. Considering the overlapping patient age for the onset of these diseases, caution is warranted in the care for RA to address the potential occurrence of TB.

Patients with RA often use immunosuppressive agents, such as disease-modifying antirheumatic drugs (DMARDs) including methotrexate, biologics, and Janus kinase (JAK) inhibitors. Confirming the presence of TB infection before initiating treatment with these drugs is essential. Previous reports suggested that individuals using immunosuppressive agents are at risk of developing TB, emphasising the importance of early detection and treatment of LTBI to prevent reactivation of TB [[4], [5], [6]].

The assessment of TB infection involves a comprehensive evaluation of results obtained from interviews, symptom recording, chest X-ray analysis, chest computed tomography scans, interferon-gamma release assay (IGRA) tests, tuberculin skin tests, and acid-fast bacilli tests. Because the Bacillus Calmette-Guérin vaccination rate is high in Japan, the tuberculin skin tests may yield false-positive results. Thus, IGRA is considered a reliable diagnostic test.

The management of IGRA-positive results in actual rheumatology practice is currently unclear. Furthermore, there are a few reports on the screening, prevalence, treatment, and prognosis of LTBI for patients with rheumatic diseases [[7], [8], [9]]. Thus, based on IGRA data obtained from the rheumatology department in Japan, we sought to clarify the actual situation in this clinical setting. For this purpose, we investigated the rates of IGRA positivity for the past 10 years and analysed the risk of active TB in real-world patients. Furthermore, we conducted a long-term prognostic study with an average follow-up period of 4.9 years to determine whether active TB occurred in patients with LTBI. In addition, we examined the utility of neutrophil cluster of differentiation 64 (CD64) to discriminate active TB from LTBI in patients with positive IGRA.

## METHODS

### Patients

Patients were tested with IGRA at the Department of Rheumatology (National Hospital Organization [NHO] Sagamihara National Hospital, Sagamihara, Japan) between 2013 and 2022. The data and information of patients included in this study were anonymised. Consent for participation in this study was provided by the patients using the opt-out consent procedure in accordance with institutional policy. The analyses were approved by the ethics committee of NHO Sagamihara National Hospital (approval no. 2017-009). This study was conducted in accordance with the principles stipulated in the Declaration of Helsinki.

### IGRA

IGRA measures the levels of interferon-gamma produced by T cells sensitised to TB-specific antigens. Hence, this test is useful for detecting LTBI or active TB. The T-SPOT.TB test using the enzyme-linked immunosorbent spot method (Oxford Immunotec Ltd) has been utilised for IGRA since 2013 at NHO Sagamihara National Hospital. A standard number of isolated peripheral blood mononuclear cells (250,000 per well) are stimulated with positive control (0.0015% phytohemagglutinin), negative control, and 2 TB-specific antigens (panel A, early secreted antigenic target 6 kDa protein [ESAT-6] and panel B, 10 kDa culture filtrate protein [CFP10]); the number of T cells producing interferon-gamma is determined to assess the presence of TB infection. The assay was performed according to the instructions provided by the manufacturer. The results were defined from spot counts in each sample well as follows: positive, (panel A—nil control) and/or (panel B—nil) ≥8; negative (positive control) ≥20 and both (panel A—nil) and (panel B—nil) are ≤4: borderline (panel A—nil) or (panel B—nil) is between 5 and 7; indeterminate (nil) was ≥11 or (positive control) was <20 and both (panel A—nil) and (panel B—nil) are <4.

We collected the information of patients tested with IGRA from medical records. We compared the information between IGRA-positive and IGRA-nonpositive (including negative, borderline, and indeterminate) patients; 11.2% of the patients underwent repeated IGRA testing. For patients in whom multiple tests yielded consistent results, the date and result of the first test were used. For those in whom multiple tests yielded inconsistent results (ie, positive and nonpositive), the date and result of the first positive test were used. If multiple tests produced nonpositive results (including negative), the date and result of the first negative test were used. IGRA was performed repeatedly in most patients with borderline or indeterminate IGRA results. Active TB did not occur in any patients with borderline or indeterminate results. Patients with active TB were positive for sputum or pus smear on microscopic analyses and/or *Mycobacterium tuberculosis* culture and/or genetic analyses (Cobas TaqMan 48 Analyzer, Roche Diagnostics; TRCRapid M.TB, Tosoh Bioscience). Patients with IGRA-positive results were diagnosed with LTBI after exclusion of active TB. The information of IGRA-positive patients regarding additional examinations, prescribed medications, and prognosis was also verified through review of the medical records or telephone survey in case of absence at the time of the regular hospital visit.

### Expression of CD64 molecules on neutrophils

We measured the expression of the CD64 molecule on neutrophils as an infection marker, as previously described [[10], [11], [12]], and compared it between patients with active TB and LTBI. The neutrophil CD64 data obtained closest to the IGRA test were used, whereas those of patients with obvious infections other than active TB were excluded from the analyses.

### Statistical analysis

Statistical comparisons of continuous variables between IGRA-positive and IGRA-nonpositive patients were conducted using *t* tests. Moreover, neutrophil CD64 expression levels were also compared between patients with active TB and LTBI using *t* tests. The chi-squared test was used for dichotomous variables in comparisons between these patients. Trends of IGRA positivity were analysed using the Cochran-Armitage test for trend. Trends of patient age were analysed using the Jonckheere-Terpstra test for trend. Multivariate logistic regression analysis was conducted to explore independent associations of patient characteristics with IGRA positivity. The results were expressed as odds ratios (ORs) with 95% CIs. The *P* values of <.05 indicate statistically significant differences. Data analyses were performed using BellCurve for Excel 4.07 (Social Survey Research Information Co., Ltd).

## RESULTS

### Rates of IGRA positivity in the past 10 years

**Figure 1:**
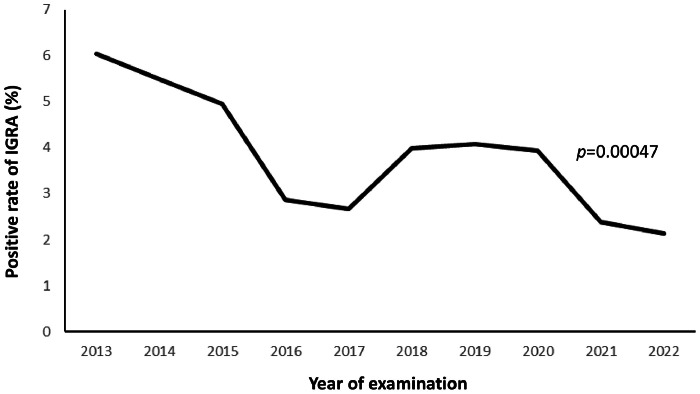
Rates of IGRA positivity in each examined year. Trends of IGRA positivity rates were evaluated using the Cochran-Armitage test for trend. IGRA, interferon-gamma release assay.

**Figure 2:**
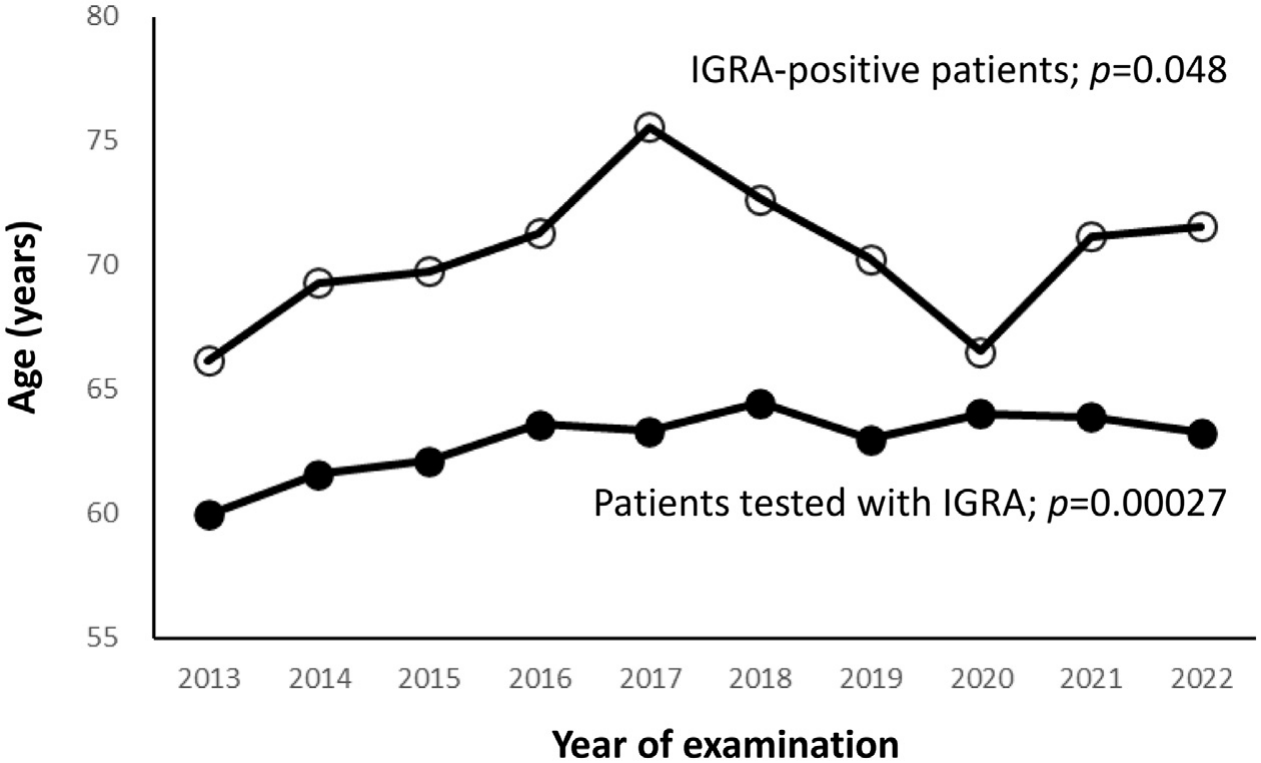
Average age of patients tested with IGRA and those with positive results in each examined year. Trends of the average ages of patients tested with IGRA were examined using the Jonckheere-Terpstra test for trend. IGRA, interferon-gamma release assay.

**Figure 3:**
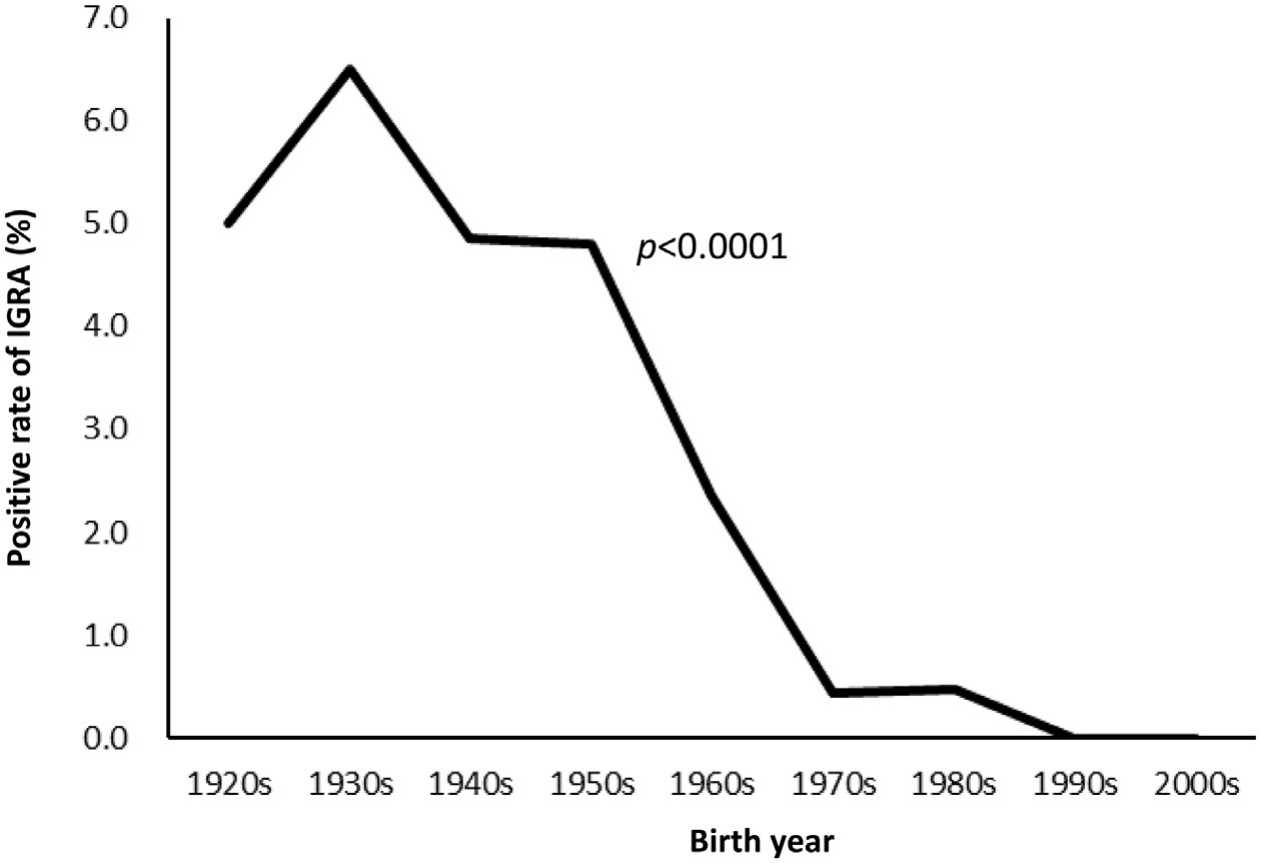
Rates of IGRA positivity according to birth year. Trends of the rates of IGRA positivity were analysed using the Cochran-Armitage test for trend. IGRA, interferon-gamma release assay.

A total of 4072 patients were tested with IGRA over a period of 10 years; positive results were obtained for 157 of those patients (positivity rate: 3.9%) (Table 1). The positive rate of IGRA decreased from 6.0% in 2013 to 2.1% in 2022 (*P* = .00047) (Fig 1), whereas the ages of IGRA-positive patients increased over the 10 years (*P* = .048) (Fig 2). Furthermore, we analysed the rate of IGRA positivity according to birth year (Fig 3). The rates of positive tests were higher among patients born before 1960 vs those born thereafter (*P* < .0001).

**Table 1:** IRGA test results

IGRA test results	Positive result	Nonpositive result		*P* value
*n* (%)	157 (3.9)	3,915 (96.1)		
		Negative	3827 (94.0)	
		Borderline	68 (1.7)	
		Indeterminate	20 (0.5)	
Age, y	70.0 ± 10.4	62.6 ± 16.0		<.0001
Female patients, *n* (%) Male patients, *n* (%)	97 (61.8) 60 (38.2)	2896 (74.0) 1019 (26.0)		<.0001

IGRA, interferon-gamma release assay.

### Characteristics of IGRA-positive patients

**Figure 4:**
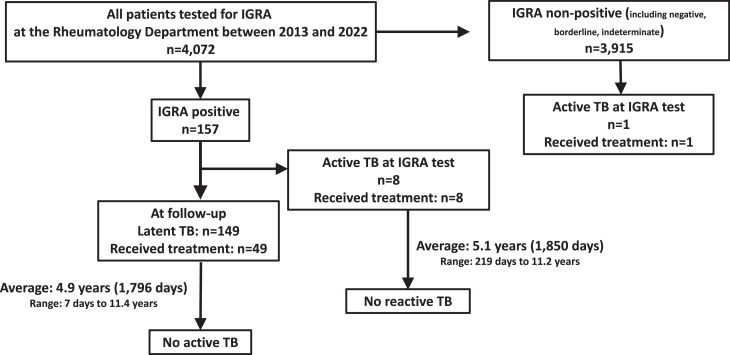
Flow chart depicting patients examined with IGRA. IGRA, interferon-gamma release assay.

Characteristics of IGRA-positive patients are shown in Figure 4. At the time of testing, 5.1% of positive patients (8/157) were diagnosed with active TB. Among the nonpositive patients (3915, 96.1%), 1 patient (0.03%) was diagnosed with active TB. Positive patients were significantly older than nonpositive patients (average age: 70.0 ± 10.4 vs 62.6 ± 16.0 years, respectively, *P* < .0001) (Table 1). The percentage of women was significantly lower among positive patients vs nonpositive patients (61.8% vs 74.0%, respectively, *P* < .0001). Multivariate logistic regression analysis showed that age (OR: 1.03, 95% CI: 1.02–1.05, *P* = 4.55 × 10^−8^) and male sex (OR: 1.67, 95% CI: 1.20–2.33, *P* = .0024) were independently associated with IGRA positivity (Supplementary Table S1).

For the 157 patients with IGRA-positive results, tests were conducted at first visit to NHO Sagamihara National Hospital (104, 62.2%), at follow-up visits (prior to the initiation of intensive treatment) (36, 22.9%), during hospitalisation examinations (13, 8.3%), and at the time of consultation from other departments or during participation in clinical trials (4, 2.5%) (Table 2). According to the medical records, 28 patients (17.8%) had a history of previous TB infection, whereas 30 patients (19.1%) did not have a history of infection; moreover, information regarding infection history was not available in the medical records for 99 patients (63.1%). Among IGRA-positive patients, 97 (61.8%), 9 (5.7%), and 24 (15.3%) patients had RA, polymyalgia rheumatica, and undifferentiated arthritis, respectively; the remaining patients had other collagen diseases and fever (suspected rheumatic diseases, infections, and other causes). Additionally, 118 (75.2%) patients were treated with glucocorticoids or DMARDs, including immunosuppressive drugs.

**Table 2:** Characteristics of IGRA-positive patients (*n* = 157)

Variable	*n* (%)
Timing of the IGRA test	
First visit	104 (62.2)
Before changing or adding treatment	36 (22.9)
Examinations during hospitalisation	13 (8.3)
Others	4 (2.5)
History of previous TB infection	
Yes	28 (17.8)
No	30 (19.1)
Unknown	99 (63.1)
Basic disease	
Rheumatoid arthritis	97 (61.8)
Polymyalgia rheumatica	9 (5.7)
Undifferentiated arthritis	24 (15.3)
Other collagen diseases	18 (11.5)
Fever (suspected rheumatic diseases, infections, and other causes)	9 (5.7)
Examinations	
Acid-fast bacilli tests	106 (67.5)
Chest X-ray examinations or computed tomography scans	152 (96.8)
No examinations	3 (1.9)
Treatment with glucocorticoids or DMARDs (including combination)	118 (75.2)
Glucocorticoids	61 (38.9)
Methotrexate	76 (48.4)
Tacrolimus	24 (15.3)
Leflunomide	5 (3.2)
Azathioprine	2 (1.3)
Sulfasalazine	60 (38.2)
Bucillamine	33 (21.0)
Iguratimod	27 (17.2)
Gold sodium thiomalate	4 (2.5)
D-Penicillamine	2 (1.3)
Auranofin	2 (1.3)
Biologics	23 (14.6)
Janus kinase inhibitors	10 (6.4)
Treatment with isoniazid	
All patients (*n* = 157)	50 (31.8)
Patients treated with glucocorticoids or DMARDs (*n* = 118)	49 (41.5)
Patients treated with biologics or Janus kinase inhibitors (*n* = 29)	23 (79.3)

DMARDs, disease-modifying antirheumatic drugs; IGRA, interferon-gamma release assay; TB, tuberculosis.

We also investigated the clinical management of those 157 patients. Acid-fast bacilli tests, including sputum or pus smear on microscopic analyses, *Mycobacterium tuberculosis* culture, and/or genetic analyses, were conducted in 106 patients (67.5%). Chest X-ray examinations or computed tomography scans were performed in 152 patients (96.8%). Of note, 104 patients (66.2%) underwent both examinations; 50 patients (31.8%) underwent 1 examination, whereas 3 patients (1.9%) did not undergo examination. Of the 157 IGRA-positive patients, 50 (31.8%) received preventive treatment for LTBI with isoniazid (INH). Of the 118 patients (75.2%) treated with corticosteroids or DMARDs, 49 patients (41.5% of the 118 patients) received preventive treatment with INH. Of the 29 patients (18.5%) treated with biologics or JAK inhibitors, 23 (79.3% of the 29 patients) received INH (Table 2).

### Survey for the prognosis of IGRA-positive patients

We assessed the diagnosis and prognosis of the 157 IGRA-positive patients (Fig 4); 8 patients were diagnosed with active TB almost concurrently. The remaining 149 patients were diagnosed with LTBI and followed up for an average of 1796 days (4.9 years) (minimum: 7 days, maximum: 4154 days [11.4 years]). During this follow-up period, none of these patients developed active TB. The 8 patients who received treatment for active TB were followed up for TB reactivation. The average follow-up period after TB treatment was 1850 days (5.1 years) (minimum: 219 days, maximum: 4091 days [11.2 years]). Recurrence of TB was not observed in these patients. Although 40.1% of IGRA-positive patients continued to visit our hospital (mean follow-up: 6.4 ± 2.9 years) and 10.8% had their status confirmed by telephone after discontinuing visits (mean follow-up: 9.1 ± 2.1 years), 8.9% died (mean follow-up: 3.8 ± 2.8 years), and 40.1% discontinued visits without telephone confirmation, including transfer to another hospital (mean follow-up: 2.6 ± 2.6 years). For these patients without telephone confirmation, the date of the last recorded clinic visit was used as the final follow-up date.

### Nine cases of active TB

The profiles of the 9 cases (IGRA positive [*n* = 8] and negative [*n* = 1]) of active TB are shown in Table 3. Of those, 4 experienced the onset of active TB between 2013 and 2014. All these patients were aged ≥50 years, and the percentage of males was 55.6% (*n* = 5). Four patients had RA and were treated with some DMARDs. Seven patients exhibited symptoms of TB, whereas 2 were asymptomatic. Low levels of serum albumin were detected in most patients at the onset of TB, suggesting undernourishment. Additionally, we measured the expression of the CD64 molecule on neutrophils. Neutrophil CD64 was measured in 5 of the 9 patients with active TB. All 5 patients showed markedly elevated neutrophil CD64 levels vs 60 patients with LTBI (mean ± SD: 14,771.8 ± 6176.9 vs 1125.0 ± 427.0, respectively, *P* = .0078) (Supplementary Fig S1).

**Table 3:** Nine cases with active TB

Case number	1	2	3	4	5	6	7	8	9
IGRA	Positive	Positive	Positive	Positive	Positive	Positive	Positive	Positive	Negative
Year of onset	2013	2013	2014	2014	2016	2018	2019	2021	2021
Sex	Male	Female	Female	Male	Male	Female	Female	Male	Male
Age, y	60	69	68	89	69	75	80	71	51
Diseases	RA	Old cerebral infarction, ovarian tumour	RA	Old cerebral infarction, dementia	Old cerebral infarction, diabetes	RA	Zoster, diverticulitis, glaucoma	RA	COPD
Symptoms at the time of TB onset	No symptom (on screening)	No symptom (on screening)	Fever, cough	Fever	Fever, dyspnoea	Fever, anorexia	Fever, cough, sputum	Dyspnoea	Fever, cough, sputum
Pulmonary or extrapulmonary	Pulmonary	Pulmonary	Pulmonary and intestinal	Spine	Pulmonary and spine	Pulmonary	Pulmonary and pleurisy	Pulmonary	Pulmonary
DMARDs	MTX, PSL	None	Certolizumab	None	None	MTX, PSL	None	IGU, SASP	None
History of TB or contact with a patient with TB	Unknown	Contact with TB	Unknown	Unknown	None	Unknown	History of TB (18 years old)	Unknown	Unknown
Alb (g/dL) at TB examinations	3.3	4.1	3 (with dehydration)	2.9	2.6	2.7	3.7 (with dehydration)	2.5	2.4
CD64 (max) (molecules/cell) (cutoff value <2000)	No data	No data	9627	15,315	7717	18,582	No data	No data	22,618

Alb, albumin; CD64, cluster of differentiation 64; COPD, chronic obstructive pulmonary disease; DMARDs, disease-modifying antirheumatic drugs; IGRA, interferon-gamma release assay; IGU, iguratimod; MTX, methotrexate; PSL, prednisolone; RA, rheumatoid arthritis; SASP, sulfasalazine; TB, tuberculosis.

## DISCUSSION

This study investigated the positive rate of IGRA over the long term. It also represents the first analysis based on long-term follow-up data regarding the clinical management of patients with IGRA-positive results in rheumatology. Our results are consistent with those of previous studies on IGRA positivity in general populations in Japan [[13], [14], [15]]. The present study showed that the rates of IGRA positivity gradually decreased with time. Additionally, this rate sharply declined among those born after 1970. These decreasing trends might reflect the decreasing rates of IGRA positivity based on birth year in Japan [[13], [14], [15]]. The health insurance system in Japan covers the administration of biologics and JAK inhibitors in elderly patients with RA. Furthermore, the proportion of elderly patients with RA is increasing in Japan. Thus, the average age of IGRA-positive patients in each examined year increased.

Importantly, >90% of the IGRA-positive patients had rheumatic diseases, and >90% of those patients underwent either bacterial or imaging tests. Eight patients had active TB, whereas the remaining 149 patients had LTBI. We investigated the prognosis of patients with LTBI; there was no case of active TB onset observed during the average follow-up period of 4.9 years. This finding indicated that IGRA-positive patients should undergo bacterial or imaging tests to rule out the possibility of active TB and be monitored through long-term follow-up.

Prior to the administration of biologics or JAK inhibitors, it is recommended that patients at high risk of developing TB receive prophylactic treatment with INH and/or rifampicin [[4], [5], [6]]. In our study, based on these recommendations, individual physicians assessed the risk in each patient and decided to initiate prophylactic treatment accordingly. Consequently, 31.8% of IGRA-positive patients (50/157) received preventive treatment with INH, and 41.5% of patients (49/118) treated with corticosteroids or DMARDs received INH. Among the 29 patients treated with biologics or JAK inhibitors, 23 (79.3%) underwent preventive therapy. Although prophylactic drugs were administered in 79.3% of patients receiving biologic or targeted synthetic DMARDs, there was no occurrence of active TB during the observation period. Of note, the sample size of the study and the follow-up period may be insufficient, and the possibility of selection bias cannot be ruled out. These data suggest that prompt evaluation of IGRA-positive patients for the development of active TB is important and that high-risk patients should be followed up for a longer period of time.

Nine patients (IGRA-positive: *n* = 8; IGRA-negative: *n* = 1) had active TB. Low serum albumin levels were observed in most patients at the onset of TB, suggesting that malnutrition is a risk factor for the development of active TB. Moreover, 2 patients with active TB did not exhibit symptoms at routine screening. This observation emphasises the importance of TB screening, including actively conducting CT scans for high-risk individuals, before the initiation of treatment with DMARDs. One case of active TB (IGRA-negative patient) had chronic obstructive pulmonary disease, did not use immunosuppressants, and might have been in the early stage of infection.

The expression of the CD64 molecule on neutrophils is upregulated by various infections and is notably higher in patients with active TB than in those with other infections [11,12]. CD64 expression is also higher among patients with RA with infection than in those without infection and is not affected by the disease activity of RA [11]. Neutrophil CD64 levels of patients with active TB were markedly elevated than those of patients with LTBI. It was also previously reported that CD64 reflected disease activity in the clinical course of patients with active TB [12]. CD64 may be used as an adjunct to evaluate the activity of TB. Moreover, examining CD64 is recommended for individuals at high risk of TB.

This study had certain limitations. Firstly, this was a retrospective investigation, and the analysis was based on information collected from medical records; therefore, the collected data may be insufficient in some cases. Secondly, we were not able to thoroughly review the medical records of IGRA-negative patients. Finally, the CD64 expression levels were determined in only 5 patients with active TB.

The rate of IGRA positivity decreased over a period of 10 years. However, this rate increased with patient age. The rates of IGRA positivity were higher among patients born before 1960. These data suggested that the incidence rates of LTBI decreased in this rheumatology department, whereas they increased in older patients.

Mycobacterial or imaging tests were performed in almost all IGRA-positive patients registered in the department of rheumatology. Although less than one-third of the 157 IGRA-positive patients were treated with INH, there was no development of active TB in these patients. This result highlighted the importance of performing mycobacterial and imaging tests in such patients, as well as continuing follow-up for extended periods of time.

## CRediT authorship contribution statement

**Shinichi Nogi:** Writing – review & editing, Writing – original draft, Validation, Formal analysis, Data curation, Conceptualization. **Hiroshi Furukawa:** Writing – review & editing, Conceptualization. **Yusuke Yano:** Writing – review & editing. **Shigeto Tohma:** Writing – review & editing, Conceptualization. **Atsushi Hashimoto:** Writing – review & editing. **Toshihiro Matsui:** Writing – review & editing.

## Competing interests

The authors state that they have nothing to declare

## Appendix Supplementary materials

Image, application 1

Image, application 2

## Footnotes

Supplementary material associated with this article can be found in the online version at doi:10.1016/j.ero.2025.10.003.
