# Supplementary figures and images for: How should we manage patients with interferon-gamma release assay-positive results in rheumatology: assessing the risk and long-term prognosis of tuberculosis
Source: EULAR Rheumatol Open. 2025 Nov 5;1(4):361–7. doi: 10.1016/j.ero.2025.10.003 (PMC13292544; doi:10.1016/j.ero.2025.10.003)

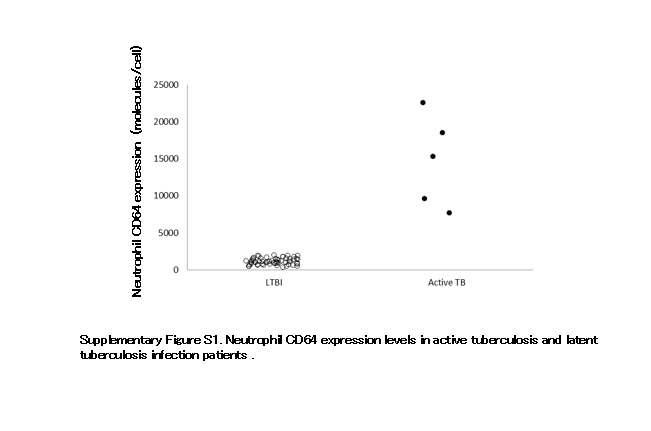

Supplement: Supplementary file 1 [file mmc1.docx]
