# Supplementary material for: How should we manage patients with interferon-gamma release assay-positive results in rheumatology: assessing the risk and long-term prognosis of tuberculosis
Source: EULAR Rheumatol Open. 2025 Nov 5;1(4):361–7. doi: 10.1016/j.ero.2025.10.003 (PMC13292544; doi:10.1016/j.ero.2025.10.003)
Supplement: Supplementary file 2 [file mmc2.pdf]

Table S1. Multivariate logistic regression analysis of clinical manifestations for IGRA.

	Unconditioned			Conditioned on the other factors		
	OR	95%CI	P	$OR_{adjusted}$	95%CI	$P_{adjusted}$
Age, years	1.04	(1.02-1.05)	$2.11X10^{-8}$	1.03	(1.02-1.05)	$4.55X10^{-8}$
Male	1.76	(1.26-2.44)	0.0008	1.67	(1.20-2.33)	0.0024

IGRA: interferon-gamma release assay, OR: odds ratio, CI: confidence interval. P, OR, 95%CI, $P_{adjusted}$, $OR_{adjusted}$ were calculated by logistic regression analysis.
